# Double Trouble: Visual and Phonological Impairments in English Dyslexic Readers

**DOI:** 10.3389/fpsyg.2019.02725

**Published:** 2019-12-17

**Authors:** Serena Provazza, Anne-Marie Adams, David Giofrè, Daniel John Roberts

**Affiliations:** ^1^Natural Sciences and Psychology, Liverpool John Moores University, Liverpool, United Kingdom; ^2^Department of Educational Sciences, University of Genoa, Genoa, Italy; ^3^Division of Psychology, Centre for Cognitive Neuroscience, College of Health and Life Sciences, Brunel University London, Uxbridge, United Kingdom

**Keywords:** developmental dyslexia, dual-route cascaded model, triangle model, visuo-spatial working memory, visual processing

## Abstract

Developmental dyslexia is a reading disorder characterized by problems in accurate or fluent reading. A deficiency in phonological processing is thought to underpin the reading difficulties of individuals with developmental dyslexia and a variety of explanations have been proposed including deficits in phonological awareness and verbal memory. Recent investigations have begun to suggest that developmental deficits in the acquisition of reading may also co-occur with visual processing deficits, which are particularly salient for visually complex stimuli, yet these deficits have received relatively little attention from researchers. To further explore the nature of phonological and visual processing in developmental dyslexia, we administered a series of non-reading tasks tapping both domains. Unsurprisingly, individuals with developmental dyslexia performed worse than typically developing readers in phonological tasks. More intriguingly, they also struggled with visual tasks, specifically when discriminating between novel visual patterns, and in visuo-spatial working memory, which requires greater attentional control. These findings highlight that individuals with developmental dyslexia present not only with phonological impairments but also difficulties in processing visual materials. This aspect has received limited attention in previous literature and represents an aspect of novelty of this study. The dual phonological and visual impairments suggest that developmental dyslexia is a complex disorder characterized by deficits in different cognitive mechanisms that underpin reading.

Developmental dyslexia (DD) is a neurodevelopmental disorder characterized by difficulties in reading aloud despite normal intelligence and adequate instruction (American Psychiatric Association, 2013). The cognitive basis of DD is thought to be a phonological deficit and, sometimes, this is proposed as the unique cause (Bruck, 1992; Swan and Goswami, 1997; Ramus, 2001; Vellutino, 2004). This view is widely accepted, and underpins one of the primary models explaining the reading disorder in DD, the Dual-Route Cascaded model, DRC (Coltheart et al., 2001).

In this model, reading is assumed to involve two major processes, or “routes” (Figure 1). First, one can access stored word pronunciations in the phonological lexicon following activation from the orthographic lexicon or semantic system. This lexical process is necessary when reading words with ambiguous or irregular spellings such as *colonel*. Second, reading can occur *via* a sub-lexical grapheme-to-phoneme conversion process. In contrast to the lexical process, the sub-lexical process can generate plausible pronunciations for regular words or phonologically plausible non-words but will produce regularization errors for irregular words (e.g., *colonel →* “colernel”; *yacht →* “yatched”; *sugar →* “sudger”).

**Figure 1:**
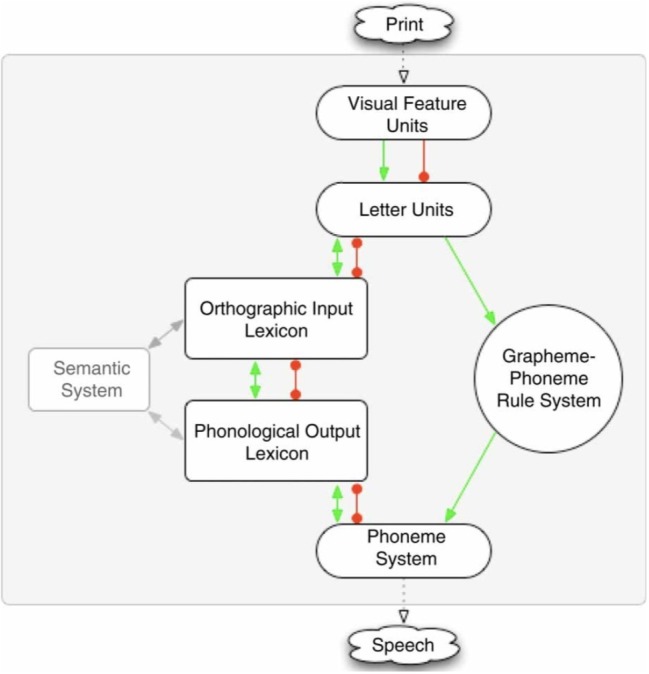
The DRC model (Coltheart et al., 2001).

Although several subtypes of DD have been described (Castles and Coltheart, 1993; Castles et al., 2006; Friedmann and Coltheart, 2016), there are two strands of evidence that point to some degree of independence between lexical and sub-lexical routes (Castles et al., 2006). First, developmental surface dyslexia is characterized by a difficulty in reading irregular words due to a deficit in the lexical route (Job et al., 1984; Castles, 1996; Zoccolotti et al., 1999). Second, phonological dyslexia is characterized by a difficulty in reading unfamiliar words or non-words due to a deficit in the phonological or sub-lexical route (Temple and Marshall, 1983; Snowling and Hulme, 1989; Rack et al., 1992).

Despite evidence indicating that impaired phonological processing represents the core deficit in DD, which may lie within these linguistic routes, there is little consensus regarding the specific mechanisms underlying lexical and sub-lexical processes and the heterogeneity of the difficulties presented by individuals with DD. In fact, there is evidence demonstrating that DD may also be characterized by a deficit in different domains, such as auditory and visual processing.

Many studies have demonstrated that some individuals with DD may present with an auditory deficit. For instance, impaired processing of brief sounds can affect speech perception in these cases (Tallal, 1980; Wright et al., 1997; Heiervang et al., 2002; Rey et al., 2002; Fraser et al., 2010; Molinaro et al., 2016). Auditory deficits are somewhat independent from phonology, but nevertheless play a role in the severity of the observed phonological deficit (see Ramus, 2003 for an extensive literature review on this aspect).

More striking evidence of the heterogeneity of DD comes from studies that show not all individuals with DD manifest phonological impairments (Bosse et al., 2007; Jones et al., 2008; Lobier et al., 2012). These findings raise the interesting possibility that different performance patterns might actually reflect distinct underlying mechanisms, rather than differences in the processes or relationships within and between the two routes (Stein and Fowler, 1981; Valdois et al., 2003, 2004; Lobier et al., 2012; Stein, 2018).

Furthermore, individuals with DD may struggle to process visual stimuli, with some presenting with dysfunction in visuo-spatial attention (Stein and Walsh, 1997; Vidyasagar and Pammer, 2010). This is often present with an asymmetrical distribution of spatial attention in the two visual fields, such that one is unable to inhibit information from the right visual field and focus attention in the center of gaze (e.g., Martin and Lovegrove, 1984; Boden and Giaschi, 2007). This pattern of difficulties may disrupt allocation of attention across letters and is generally attributed to a deficit of the magnocellular pathway, and in particular the dorsal pathway, which is involved in the analysis of motion perception (see e.g., Ramus, 2003 for a review of the magnocellular deficit in DD). However, despite evidence showing that the magnocellular deficit can contribute to DD, it remains a controversial and hotly debated issue. In fact, there is no clear evidence that a deficit in the magnocellular pathway can contribute to the reading difficulties in DD, independently of phonological impairments. Moreover, a deficit in the magnocellular pathway has been reported in the scenario of phonological dyslexia but not in surface dyslexia, leaving open the question of what causes this subtype of dyslexia (see, e.g., Spinelli et al., 1997; Valdois et al., 2004).

Theories explaining deficits in visuo-attention span underpinning surface dyslexia have contributed to the debate with the most prominent of these being work by Bosse et al. (2007). The visuo-attention span hypothesis posits that difficulties in DD are a consequence of a deficit in visual processing. In this vein, the visuo-attention span hypothesis underpins the existence of a visual system impairment in this population (Lobier et al., 2012; Zoubrinetzky et al., 2016; Frey and Bosse, 2018).

The visuo-attention span theory is derived from the connectionist multi-trace memory model of reading (Ans et al., 1998) and postulates the existence of two reading procedures that are characterized by different “attentional windows” – the analytic (serial) and global (parallel) procedures. The analytic mode uses a narrow attentional window or “spotlight” which serially processes orthographic sub-units (letters or letter combinations) within the word. During this mode, phonological outputs corresponding to each sub-unit are generated successively and have to be maintained in a buffer (memory trace) for phonological production. The global mode uses a wide attentional window or “floodlight” which permits automatic recognition or parallel processing of the whole word during reading aloud, and thus generates the entire phonological output without involvement of the buffer. In this framework, visual attention span is defined as the number of elements (letter units) processed simultaneously (Lallier and Valdois, 2012).

Familiar words are generally processed through the global mode, employing a wide visual attentional window, whereas unfamiliar words are processed through the analytic mode, employing a narrow attentional window – this is because more attention is needed to generate phonological representations from a combination of unfamiliar letter units. If the normal visual attention span is reduced, reading becomes reliant on the analytic mode. Due to the inability of this mode to generate the entire output, irregular word reading becomes slow and regularization errors may occur. Hence, this theory is able to account for word recognition difficulties in one sub-group of individuals with DD independently of phonology, conceptualized as surface dyslexia, whereas word recognition difficulties in individuals with phonological dyslexia might be better captured by phonological deficits (Valdois et al., 2003; Bosse et al., 2007; Lobier et al., 2012; Zoubrinetzky et al., 2014, 2016; Stefanac et al., 2019).

Taken together, these findings support the claim that individuals with DD may present with impairments in the visual domain that are not restricted to word processing. Studies investigating visual processing of non-orthographic stimuli (e.g., faces and objects) have indeed demonstrated atypical performance, which strengthens the hypothesis that a visual impairment may characterize some types of DD (Sigurdardottir et al., 2015, 2018; Gabay et al., 2017; Robotham and Starrfelt, 2017). Such evidence cannot be fully accounted for by lexically based visual word recognition models (i.e., the DRC model).

An alternative approach that might accommodate some of these findings proposes that disorders of reading do not occur in isolation but are an emergent effect of damage to one of three primary systems (vision, phonology, semantics), or damaged input to them, to which reading may be more susceptible (Patterson and Lambon Ralph, 1999; Hoffman et al., 2015). The triangle model (Figure 2) is an instantiation of the primary systems hypothesis, and proposes that the same computational elements, in various combinations, support different reading and non-reading activities: (1) vision, which with respect to reading mediates knowledge about orthographic word form but also processes non-orthographic visual stimuli; (2) phonology – the internal representation of word sound, also utilized by any form of verbal input or output including naming and repetition; and (3) semantics – word meaning.

**Figure 2:**
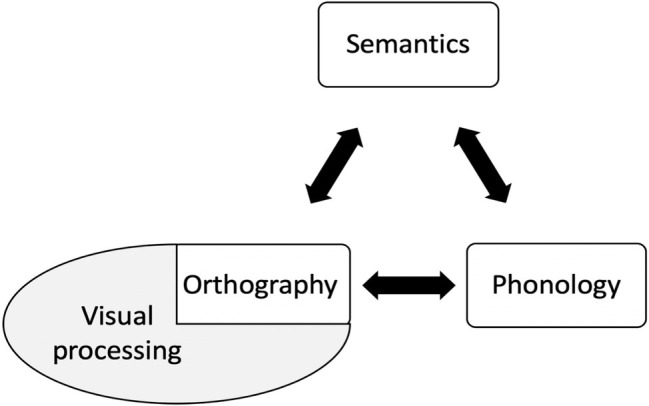
The triangle model (Seidenberg and McClleland, 1989; Plaut, 1996).

Reading is accomplished by the division of labor between the three systems. In particular, a mapping between vision and phonology (V > P) permits reading of regular words with a high speech-sound correspondence (e.g., *mint*) or high-frequency irregular words (e.g., *have*), whereas irregular words with a less regular speech-sound correspondence (e.g., *colonel*, *pint*) are supported by the semantic system (V > S > P) (Woollams, 2013).

Despite evidence confirming this alternative view in accommodating the impairments in some of the acquired dyslexias (e.g., visual impairments in pure alexia, semantic impairments in surface dyslexia, and phonological impairments in phonological dyslexia), there is insufficient evidence to establish the capacity of this model to also explain impairments in DD (Woollams, 2013). A prediction that follows from this model is that individuals with DD, when tested appropriately, will show deficits in non-reading tasks, depending on which primary system is damaged. For instance, a degraded incoming visual signal caused by a narrow attentional window will affect reading and other visual tasks that demand similar processing (Gabay et al., 2017). Moreover, due to the interactive nature of the model, it is also predicted that a dysfunctional visual system may affect performance of other types of task that necessitate visual processing such as visuo-spatial working memory.

Working memory (WM) is a limited capacity system, which enables the temporary storage and maintenance of information (Baddeley and Hitch, 1974; see Cornoldi and Giofrè, 2014 for a review). Several WM models are available but the classical tripartite model, which distinguishes between two slave systems (verbal and visuo-spatial) and a central executive component, has received substantial support in the literature (Baddeley, 1986; Cornoldi and Giofrè, 2014). Deficits in the verbal component seem to be quite severe and a core feature of performance in DD (e.g., Peng and Fuchs, 2016; Toffalini et al., 2017). Hence, previous studies have focused on the verbal domain (e.g., Majerus and Cowan, 2016), with the visuo-spatial component receiving little attention (with some exceptions see Smith-Spark et al., 2003; Smith-Spark and Fisk, 2007; Cowan et al., 2017). It therefore remains to be determined whether, as with acquired disorders of reading, visuo-spatial working memory (VSWM) impairments can explain an additional portion of the variance in word reading in DD after controlling for verbal WM skills.

According to the primary system hypothesis, a number of predictions can be made. First, individuals with DD should also present with visual deficits, as evidenced in patients with acquired dyslexia (i.e., pure alexia, see Roberts et al., 2013, 2015). To test this prediction, we used visual discrimination tasks with unfamiliar objects – checkerboards and Kanji characters (see “Methods” section and Figures 3, 4 for detailed information of these tasks). We chose to use non-orthographic stimuli to assess visual processing *per se* and to avoid underestimating the severity of the visual impairment. For instance, using familiar stimuli might result in top-down semantic support, which may compensate for, or boost activation of, an impaired visual system (Plaut, 1999). Moreover, studies conducted on DD children in Japan, a logographic orthography in which the orthographic units are pictograms (i.e., Kanji), showed that these children exhibited difficulties in reading and writing Kanji (e.g., Uno et al., 1995; Kaneko et al., 1997). The authors argued that such difficulties might be explained by problems in visual or visuo-spatial processing. Indeed, the role of phonology is less prominent in orthographies in which the units employed to read are coarser than the single grapheme (i.e., pictograms, see Wydell and Butterworth, 1999). Thus, the difficulties shown by individuals with DD in those orthographies might be underpinned by a compromised visual system. What we aim to investigate in this study is the extent to which an impairment in visual processing may also characterize DD reading of alphabetic orthographies.

**Figure 3:**
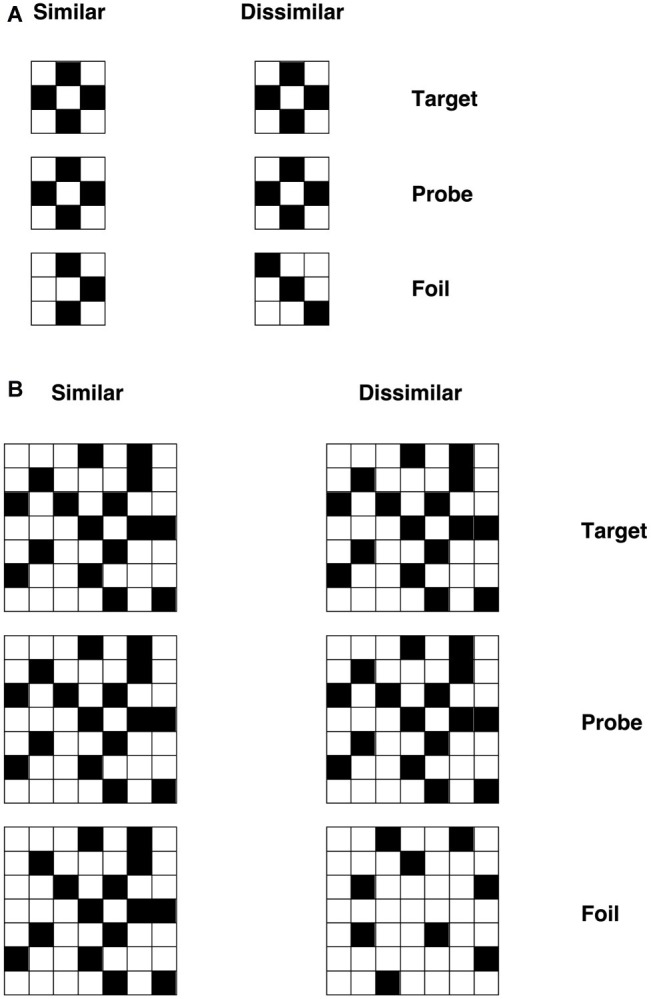
Example checkerboard stimuli for **(A)** visually simple condition and **(B)** visually complex condition with similar and dissimilar foils (Roberts et al., 2013).

**Figure 4:**
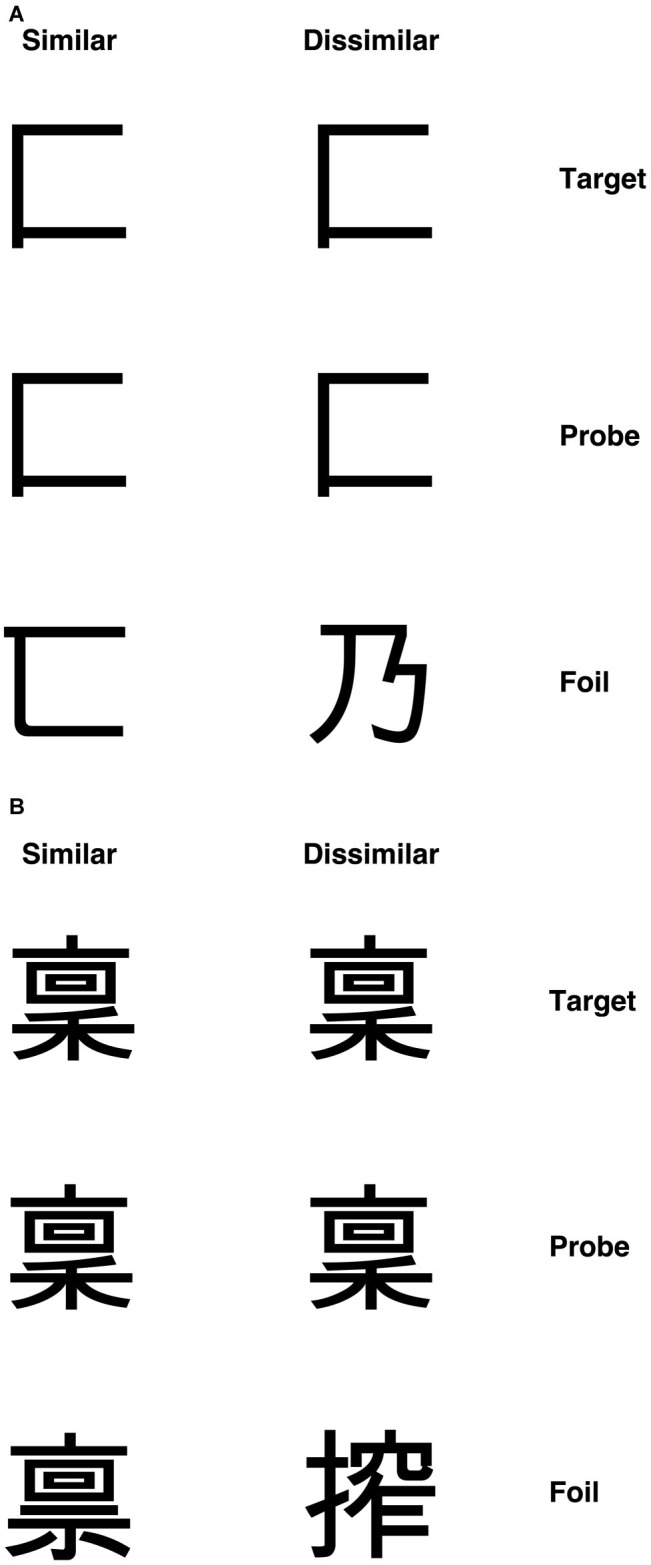
Example Kanji stimuli for **(A)** visually simple condition and **(B)** visually complex condition with similar and dissimilar foils (Roberts et al., 2013).

Second, impairments in WM are not only limited to the verbal domain but can also affect the visuo-spatial aspects, in particular those that place maximal demands on attentional control. For this reason, we used VSWM tasks that were demanding in terms of attentional control (see “Methods” section and Figure 5). We expected that (1) a low-level impairment in visual processing will affect the performance of individuals with DD in VSWM tasks and (2) poor performance will be exaggerated in tasks that require more attentional control.

**Figure 5:**
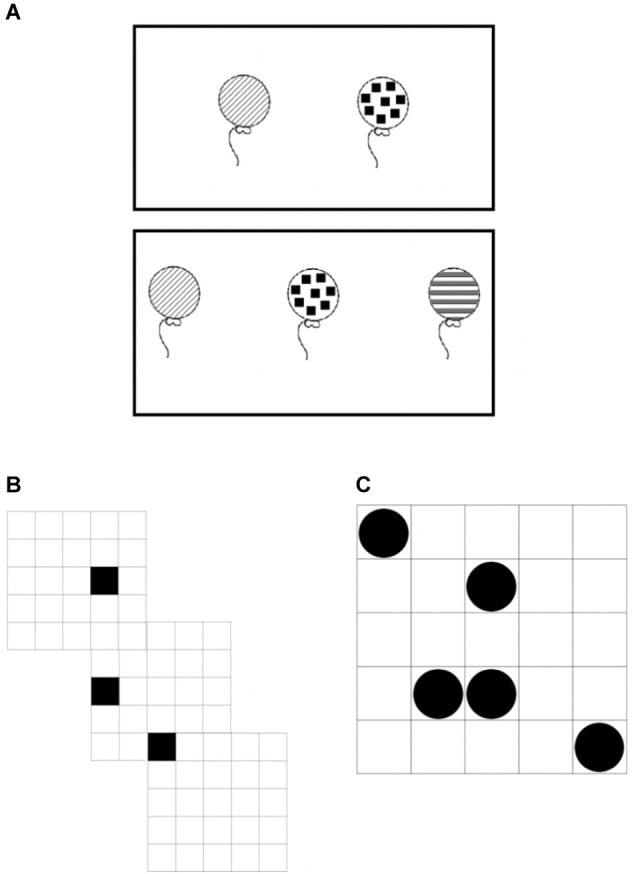
Example VSWM tasks for **(A)** balloons, **(B)** sequential matrices, and **(C)** simultaneous matrices.

Third, DD should be considered as a complex disorder encompassing general processing deficits in both phonological and visual domains. Hence, phonology was measured in accordance with the predominant literature indicating deficits in this skill in individuals with DD. Finally, DD could be also characterized by a low-level auditory deficit. Indeed, some research has shown that impaired processing of brief sounds might be detrimental to speech perception, thus aggravating the phonological deficit (e.g., Wright et al., 1997; Molinaro et al., 2016).

## Methods

### Participants

Eighteen university students with DD (five males; age range 19–27; *M*_age_ = 21.8; SD = 2.29) participated. All were native speakers of English and in receipt of a formal diagnosis of dyslexia (supplied by a registered assessor of SpLD), as required for access arrangements and additional support in UK higher education institutions. Participants with DD have been contrasted to a typically developing reader (TDR) group comprising 18 students (7 males; age range 19–28; *M*_age_ = 21.8; SD = 2). The two groups did not differ statistically for gender, *χ*^2^(1) = 0.50, *p* = 0.480, Cramer’s *V* = 0.118, or age, *F*(1, 34) = 0.02, *p* = 0.878, $\eta_{p}^{2}$ = 0.001.

The reading level of the two groups was assessed using two reading tasks (word and non-word reading, see Roberts et al., 2010). As expected, participants in the two groups differed statistically with DD performing worse than TDR when reading words, *F*(1, 34) = 6.86, *p* = 0.013, $\eta_{p}^{2}$ = 0.168, and non-words, *F*(1, 34) = 7.68, *p* = 0.009, $\eta_{p}^{2}$ = 0.184. To tap general cognitive abilities, we employed a multimodal vocabulary naming test (Hoffman and Lambon Ralph, 2013; Mackenzie-Phelan and Roberts, 2018). The two groups did not differ on this measure of general cognitive ability, *F*(1, 34) = 2.55, *p* = 0.119, $\eta_{p}^{2}$ = 0.070.

### Materials

#### Visuo-Spatial Working Memory

Three VSWM tasks were employed in this study: balloons, sequential matrices, and simultaneous matrices (Mammarella et al., 2018). Two trials for each span were presented. Partial credit score was used for scoring purposes. This scoring procedure allows for a more precise estimation of the WM capacity of each individual by considering the partial recall. For example, if a participant correctly recalled 5 out 6 stimuli in the correct order in one trial the score for that trial would be 5 (see Unsworth and Engle, 2006, 2007; Giofrè and Mammarella, 2014 for the statistical rationale). For balloons and simultaneous matrices, stimuli were simultaneously presented; therefore, the order of recalling was irrelevant for this task. For the sequential matrices span, participants were required to recall the items in the right order of presentation. In this latter task, partial recall was constituted by the sum of the stimuli correctly recalled in the correct order of presentation.

##### Balloons

The stimuli were schematic drawings seen from the front. Initially, a set of two drawings is shown for 4 s. Immediately after presentation, the participant has to recognize the target drawings (by clicking on it) within a set comprising three stimuli. Then a set of three drawings was presented for the same length of time and the participant must recognize them among a total of five drawings. From there, three larger sets of drawings were also used. The set of four, five, and six target drawings were placed in groups of six, eight, and nine drawings, respectively (min possible score = 0 and max possible score = 40).

##### Sequential Matrices

Participants were asked to memorize and recall the positions of black cells that appeared for 1 s in different positions on a 5 × 5 grid. After a series of black cells had been presented, participants clicked on the locations where they had seen a black cell appear in the right order. The number of black cells presented in each series ranged from two to eight (min possible score = 0 and max possible score = 72).

##### Simultaneous Matrices

Participants had to memorize and recall the position of a number of black dots, which appeared simultaneously for 3 s on a 5 × 5 grid. All of these tasks were of increasing difficulty. Participants were presented for 1.5 s with a 5 × 5 grid. The number of black dots presented in each grid ranged from two to eight. After 3 s the initial stimulus was removed and participants were presented with a blank test matrix in which they had to click on the previously filled squares. The number of black cells presented in each series ranged from two to eight (min possible score = 0 and max possible score = 72).

#### Visual Processing Tasks

Two visual matching tasks were employed to assess visual abilities and are described below (Roberts et al., 2013). For each of these tasks, RT and accuracy data were collected.

##### Checkerboards

A set of 32 black-and-white checkerboards were used. The number of squares in each matrix was either 9 (3 × 3) or 49 (7 × 7), forming the visually simple and visually complex sets respectively. Grids were constructed by avoiding placement of blocks of the same color together or any other regularity in the patterns (that might simplify visual processing). Stimuli were used to form a triad-based matching-to-sample task, in which the probe was flanked above and below by the target and foil. The position (above/below) of target and foil was randomized. Three vertically aligned checkerboards appeared on the screen for each trial. The central checkerboard was the probe stimulus, and the participants had to decide whether the top or bottom checkerboard matched the central one (i.e., they had to identify the target), by pressing two different keys on the keyboard (“N” for the stimulus below and “Y” for the stimulus below). Each participant was required to respond as quickly and accurately as possible.

##### Kanji Characters

A set of 60 single Kanji characters were used. Visual complexity was defined in terms of the number of strokes in each character. Characters with 2–4 strokes constituted the simple items, and those with 13 strokes formed the complex set. Again, each target character appeared in a matching-to-sample triad. The probe was placed in the center with the target and foil above or below. The position of the target was randomized across trials. Three vertically aligned Kanji characters appeared on the screen for each trial. The central Kanji character was the probe stimulus, and the participants had to decide whether the top or bottom Kanji matched the central one (i.e., they had to identify the target), by pressing two different keys on the keyboard (“N” for the stimulus below and “Y” for the stimulus below). Each participant was required to respond as quickly and accurately as possible.

#### Phonological Processing Tasks

To investigate phonological processing, the digit span test was used (Wechsler, 2008). This test consists of three subtasks: digit forward, in which participants were instructed to recall as many of the digits as possible in the same order they were presented; digit backward, in which participants had to recall the digits in the reverse order; and digit sequential, which required participants to recall the digits in ascending order of magnitude. The span test score is obtained by summing up the scores in the three span conditions (see Wechsler, 2008 for more detailed information).

#### Auditory Processing Tasks

To control for the presence of an impairment in auditory processing, an auditory matching task was employed to assess auditory abilities. The design was identical to the checkerboard and Kanji tasks (Roberts et al., 2010). This tests purely auditory processing (stripped of meaning, lexical properties etc.). Three tones were presented for each trial. The last tone was the probe stimulus, and the participants had to decide whether the first or the second tone matched the last one (i.e., they had to identify the target), by pressing two different keys on the keyboard (“1” for first stimulus presented and “2” for the second stimulus presented). Each participant was required to respond as quickly and accurately as possible. RT and accuracy data were collected for the task.

### Procedure

All tasks were administered using E-Prime 2.0 software (MacWhinney et al., 2001). Students were assessed individually in a single session lasting approximately 1 h in a quiet room at Liverpool John Moores University. The study was approved by the RES Committee North West – Liverpool Central (15/NW/0461) and written consent was obtained from all participants.

## Results

Means and standard deviations for both RTs and accuracy of the two groups are displayed in Table 1.

**Table 1:** Mean and standard deviations (SD) in all tasks.

			Statistical analyses		
Task	DD	TDR	*F*(1, 34)	*p*	$\eta_{p}^{2}$
**VSWM**					
Balloons	30.61 (4.41)	33.33 (3.38)	4.32^*^	0.045	0.113
Sequential matrices	35.17 (10.57)	48.17 (8.62)	16.35^**^	0.000	0.32
Simultaneous matrices	56.61 (6.90)	61.83 (5.61)	6.20^*^	0.018	0.15
**Visual**					
Checkerboards RTs	2866.34 (853.49)	2334.52 (525.50)	5.07^*^	0.031	0.130
Kanji RTs	1826.27 (558.80)	1411.08 (331.28)	7.35^*^	0.010	0.178
Checkerboard ACC	0.94 (0.03)	0.96 (0.04)	1.90	0.177	0.053
Kanji ACC	0.95 (0.04)	0.97 (0.02)	1.81	0.187	0.051
**Phonological**					
Span test	24.20 (4.50)	31.61 (4.34)	25.18^**^	0.000	0.426
**Auditory**					
Tone test RTs	661.70 (395.60)	436.10 (141.68)	5.15	0.030	0.131
Tone test ACC	0.81 (0.11)	0.91 (0.06)	11.66^**^	0.002	0.255

*TDR, typically developing readers; DD, developmental dyslexics; RTs, reaction times; ACC, accuracy; 0.000 means that the value is 0 when approximated to the third decimal*.

* *p < 0.05*;

** *p < 0.01*.

### Data Analyses

SPSS (Version 25; IBM, 2017) was used to perform all analyses. Before conducting the discriminant function analysis, issues related to sample size and multivariate normality were addressed (Tabachnick and Fidell, 1996). The criterion that the sample size of the smallest group should exceed the number of predictors was met. Group size was equal, ensuring multivariate normality.

### ANOVAs and MANOVAs

#### Visuo-Spatial Working Memory

**Figure 6:**
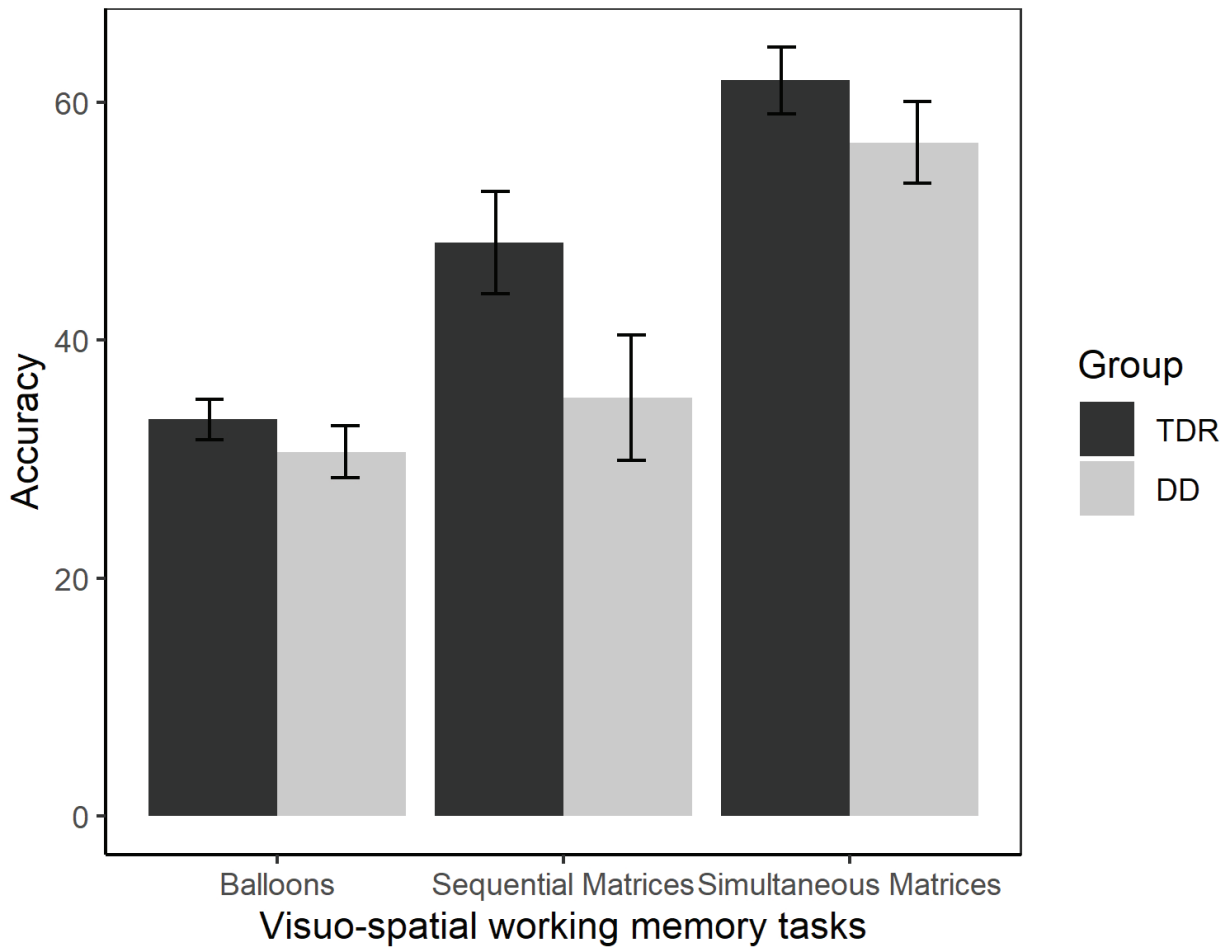
Accuracy in VSWM tasks. Error bars represent standard errors. TDR, typically developing readers, DD, developmental dyslexics.

A MANOVA was performed comparing VSWM tasks (balloons, sequential matrices, simultaneous matrices) in the two groups (Figure 6). A significant effect of group, *F*(3, 32) = 6.62, *p* = 0.001, $\eta_{p}^{2}$ = 0.383, was found, with a large effect size. Participants with DD performed significantly worse than TDR in all the VSWM tasks, with effect sizes ranging from moderate to large. Follow-up ANOVAs are presented in Table 1.

#### Visual Processing

**Figure 7:**
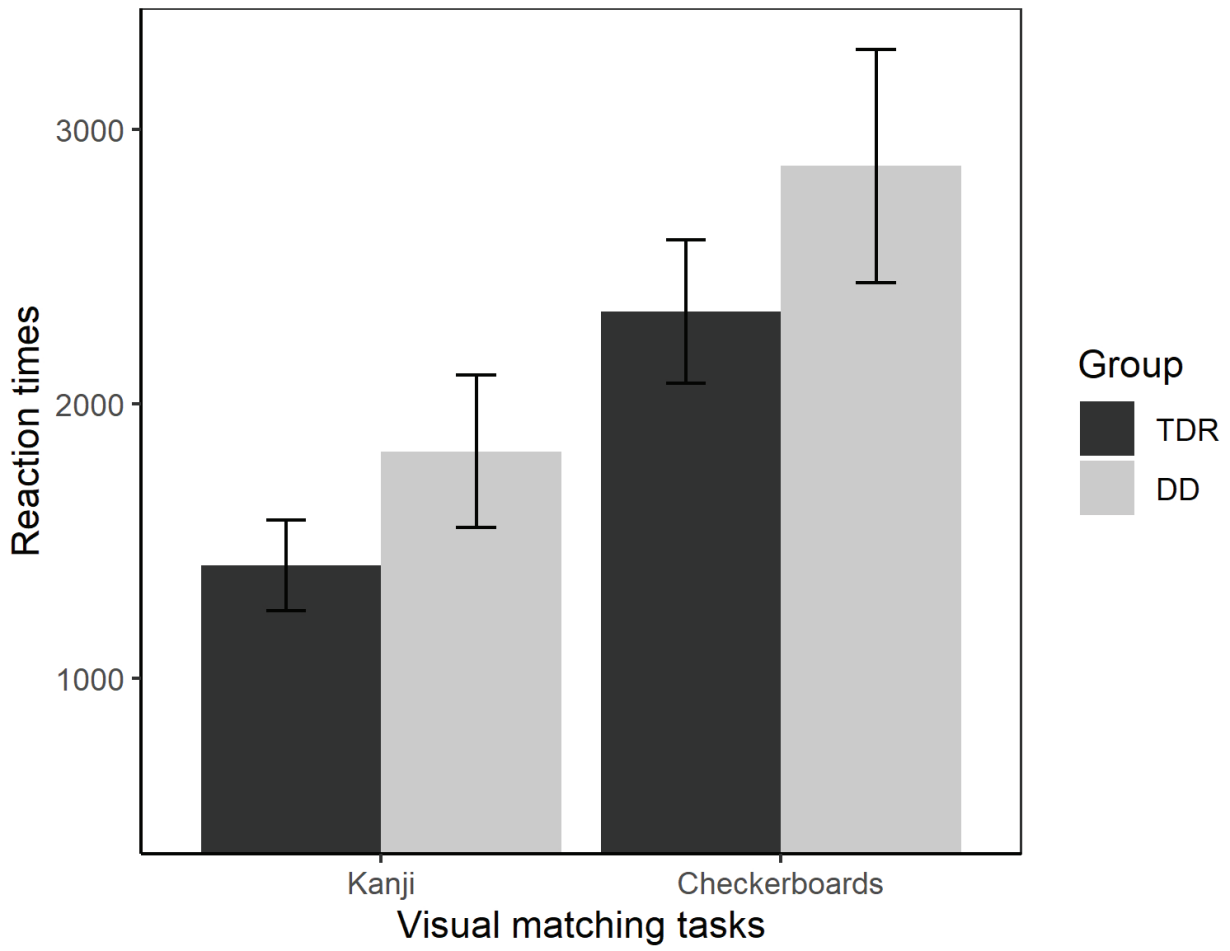
Reaction times in visual tasks. Error bars represent standard errors. TDR, typically developing readers, DD, developmental dyslexics.

A MANOVA was performed comparing RTs in the visual tasks (checkerboards and Kanji – Figure 7). A significant effect of Group, *F*(2, 33) = 3.63, *p* = 0.037, $\eta_{p}^{2}$ = 0.181, with a medium effect size was identified. Participants with DD performed worse than the TDR group in both tasks, with moderate effect sizes. A MANOVA was also performed for the accuracy in these tasks. The results showed no significant differences between the DD group and TDR group, *F*(2, 33) = 1.11, *p* = 0.340, $\eta_{p}^{2}$ = 0.063, with a small effect size. Follow-up ANOVAs are presented in Table 1.

Means and standard deviations for the four conditions in checkboards and Kanji tasks (i.e., high complexity low similarity, high complexity high similarity, low complexity high similarity, and low complexity low similarity) are presented in Table 2.

**Table 2:** Mean (*M*) and standard deviations in four conditions of the checkerboards and Kanji tasks.

	DD		TDR	
Task	*M*	SD	*M*	SD
**Checkerboards**				
*Accuracy*				
High complexity and low similarity	0.98	0.03	1.00	0.01
High complexity and high similarity	0.87	0.13	0.91	0.11
Low complexity and low similarity	0.99	0.02	0.99	0.04
Low complexity and high similarity	0.95	0.07	0.96	0.05
*Reaction time*				
High complexity and low similarity	1838.35	535.87	1417.54	344.43
High complexity and high similarity	6792.15	2630.16	5445.70	1530.99
Low complexity and low similarity	1523.55	407.04	1207.07	202.48
Low complexity and high similarity	1763.09	487.41	1428.18	249.58
**Kanji**				
*Accuracy*				
High complexity and low similarity	0.98	0.03	0.98	0.03
High complexity and high similarity	0.91	0.10	0.97	0.03
Low complexity and low similarity	0.99	0.02	0.99	0.03
Low complexity and high similarity	0.95	0.05	0.94	0.05
*Reaction time*				
High complexity and low similarity	1669.79	518.22	1299.83	282.70
High complexity and high similarity	2608.45	889.87	1932.24	541.32
Low complexity and low similarity	1204.08	328.79	952.65	171.30
Low complexity and high similarity	1912.30	714.05	1482.72	436.28

*TDR, typically developing readers; DD, developmental dyslexics*.

#### Phonological Processing

**Figure 8:**
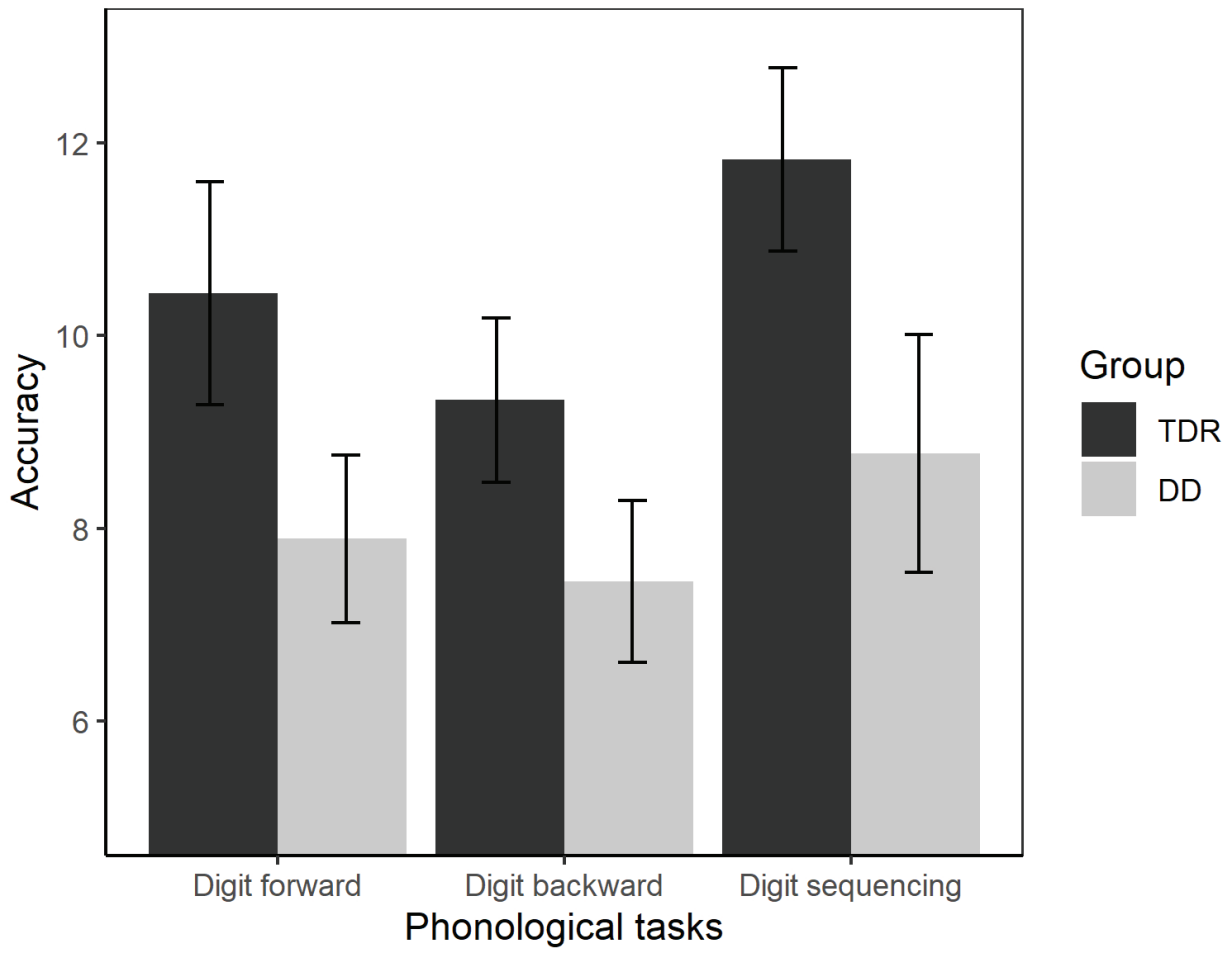
Accuracy in phonological tasks. Error bars represent standard errors. TDR, typically developing readers, DD, developmental dyslexics.

We performed an ANOVA to compare the performances of the two groups (Figure 8). The results showed that the DD group performed worse than TDR group, *F*(1, 34) = 25.18, *p* < 0.001, $\eta_{p}^{2}$ = 0.426, with a large effect size.

#### Auditory Processing

**Figure 9:**
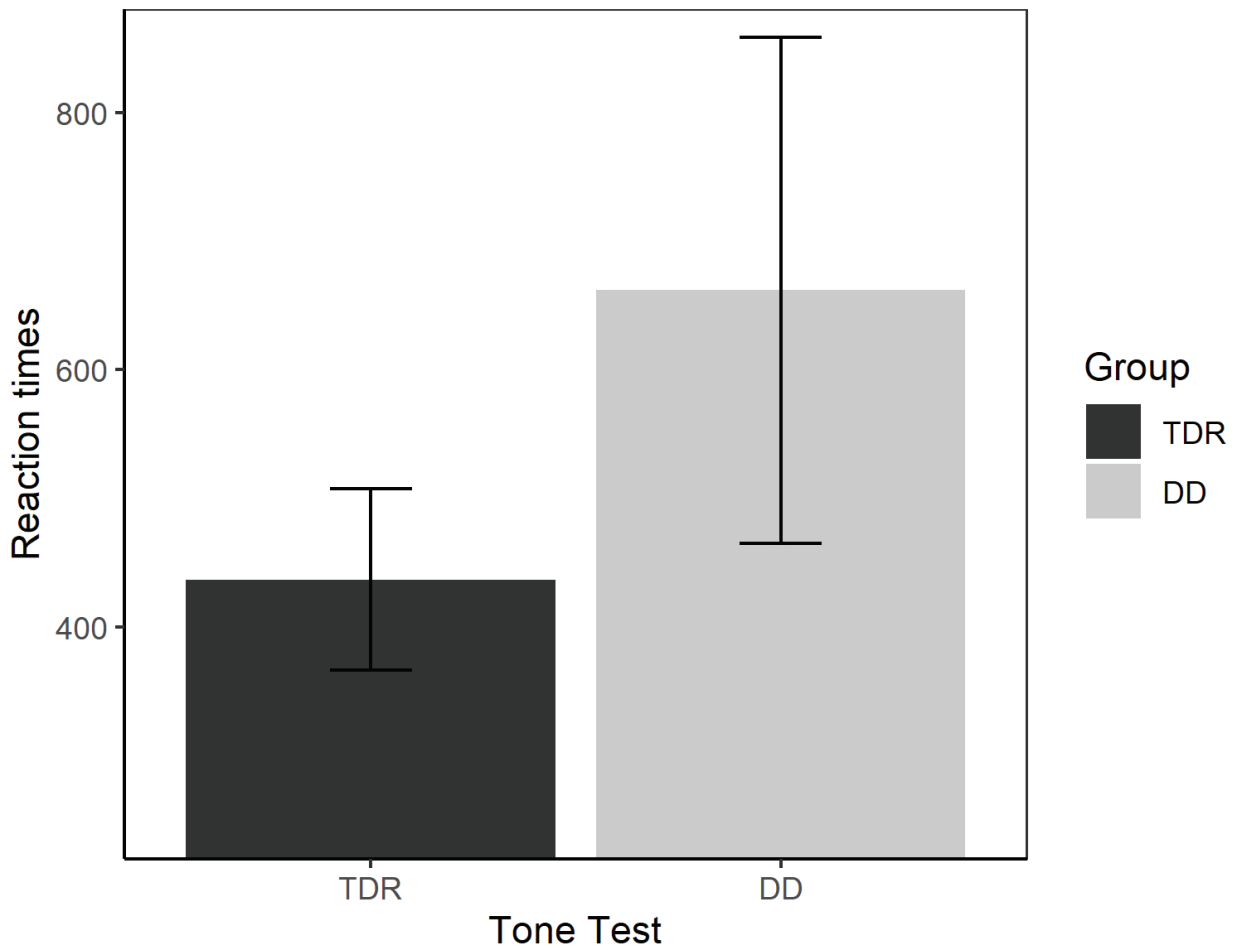
Reaction times in auditory tasks. Error bars represent standard errors. TDR, typically developing readers, DD, developmental dyslexics.

**Figure 10:**
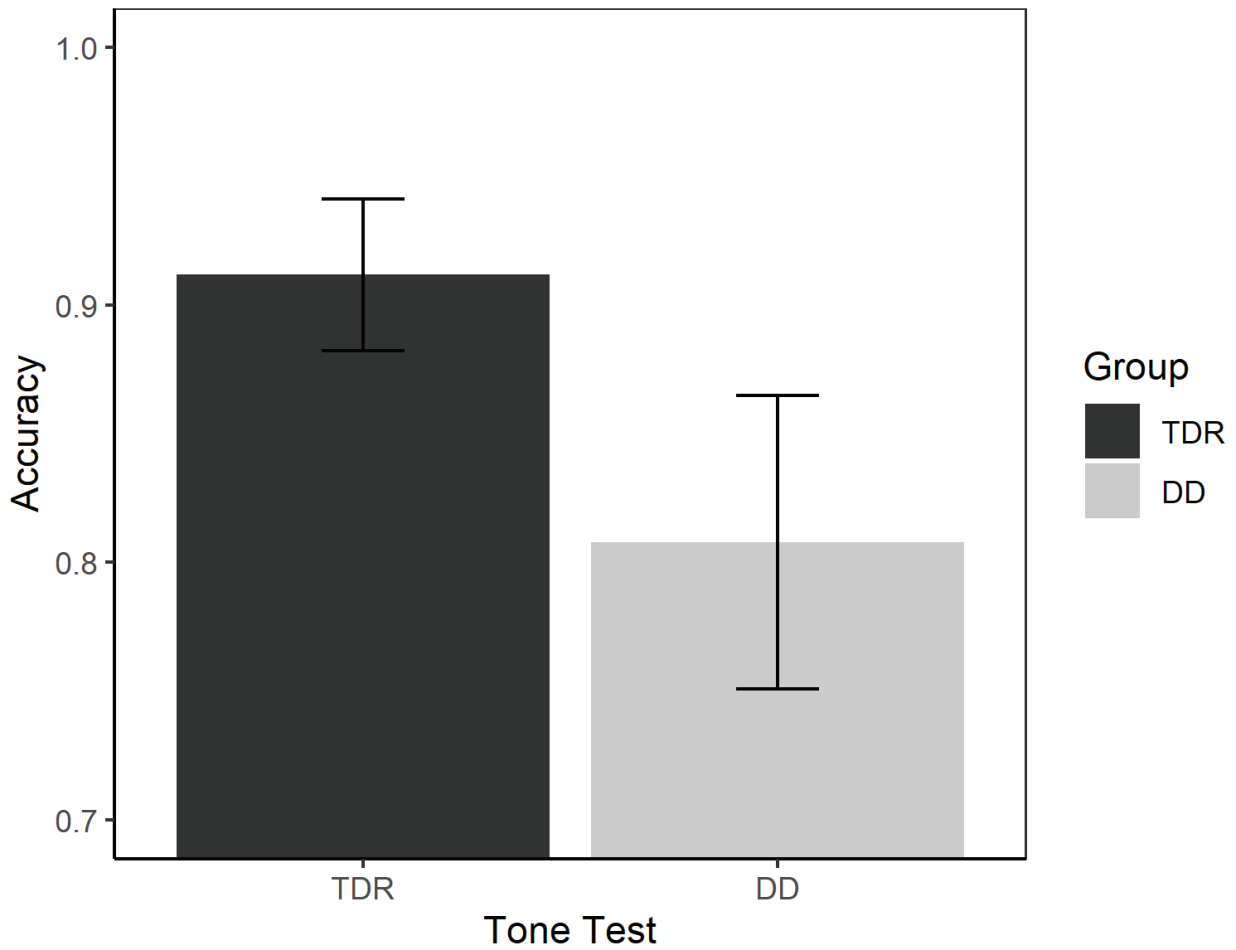
Accuracy in auditory tasks. Error bars represent standard errors. TDR, typically developing readers, DD, developmental dyslexics.

We performed an ANOVA to compare the performances of the two groups in both RTs (Figure 9) and accuracy (Figure 10). With respect to the RTs, the results showed that the DD group performed worse than TDR group, *F*(1, 34) = 5.15, *p* < 0.030, $\eta_{p}^{2}$ = 0.131, with a medium effect size. The DD group performed worse than TDR group also in accuracy, *F*(1, 34) = 11.66, *p* = 002, $\eta_{p}^{2}$ = 0.255, with a large effect size.

### Discriminant Function Analysis

Discriminant analysis is generally used to determine which variables discriminate between two or more groups. Discriminant analysis is also used to investigate how variables contribute to group separation, and to what degree. Here, discriminant function analysis was performed to establish which tasks had the greatest discriminatory power to distinguish between participants with DD and TDR. The criterion that the sample size of the smallest group should exceed the number of predictors was met, and the group size was equal, ensuring multivariate normality. The discriminant analysis was conducted with the stepwise method, using all the tasks of the VSWM, visual, verbal, and auditory domains. Only a visual matching task (i.e., the Kanji task) and a verbal working memory task (i.e., the span test) were included in the model, with a Wilk’s *λ* = 0.47, which indicates that these two predictors were the best variables to discriminate between the two groups.

The discriminant function analysis showed a reliable association with both the DD and the TDR group, *χ*^2^(2) = 25.03, *p* < 0.001. The Kanji task and the span test were able to correctly discriminate 100% of the TDR group (i.e., 18/18) and 83.3% of the DD group (i.e., 15/18). Overall, the model was able to discriminate 91.7% of the participants. This finding indicates that these two tasks, i.e., Kanji and span test, had the greater discriminatory power as compared to all the other tasks included in this study. Thus, performance on tasks from both the phonological and the visual domain is required to discriminate participants with DD from those who have made typical progress in reading.

## Discussion

The aim of this study was to investigate whether individuals with DD present with linguistic impairments only, which are well captured by the DRC model, or if they also present with impairments in the visual domain in addition to phonological impairments, a position that aligns more closely with the triangle model. We aimed to describe such impairments within the framework of the triangle model, an instantiation of the primary system hypothesis. In order to achieve these aims, a group of individuals with DD was compared with a matched group of TDRs using a comprehensive cognitive battery, including a number of visual, phonological, and auditory tasks.

The results demonstrated a phonological impairment in the DD group, who performed significantly worse on the span test, with a large effect size. This finding confirms previous evidence highlighting impaired performance compared to controls on phonological tasks (Snowling, 1981; Paulesu, 2001; Carroll and Snowling, 2004). More critically, the DD group also performed worse on VSWM and visual processing tasks. These results indicate that in addition to phonologically based deficits, individuals with DD also have difficulties in processing visual and visuo-spatial information. This has important theoretical implications, since although it is well established that individuals with DD have difficulties in verbal WM tasks (e.g., Ackerman and Dykman, 1993; Gathercole et al., 2016), evidence of a visual processing deficit, and VSWM in particular, is scarcely investigated (with some exceptions e.g., Menghini et al., 2011).

The group with DD were impaired on all VSWM tasks and disproportionately so on the sequential matrices. In fact, some visuo-spatial WM models, including for example Cornoldi and Vecchi (2003) and Kane et al. (2004), distinguished between tasks requiring different degrees of attentional control. This might explain at least in part why individuals with DD tend to be more impaired in tasks requiring sequential recall (e.g., sequential matrices and digit span), which place maximal demands on attentional control (Cornoldi and Vecchi, 2003). Notably, difficulty with sequential tasks, either verbal or visuo-spatial, also occurs in individuals with other learning difficulties, such as dyscalculia or non-verbal learning disabilities (Mammarella et al., 2013, 2018; Bizzaro et al., 2018). This supports the view that individuals with DD might struggle with sequential tasks (Plaza and Guitton, 1997; Helland and Asbjørnsen, 2003).

As for the non-orthographic visual tasks, with a minimal requirement of WM abilities, the DD group also showed an impairment, particularly in speed of responding. These findings are similar to those obtained with acquired dyslexic patients (Roberts et al., 2013) and are consistent with explanations of a visual deficit contributing to DD. It is worth noting that visual deficits were captured in speed of processing rather than accuracy, and such impairments could easily be missed if only accuracy is measured. Hence, it is important to measure response speed when visual processing is evaluated in DD and in this scenario visual impairments might be more prominent in DD. These findings, along with those obtained in the VSWM tasks, support the claim that a deficit in visual processing may characterize some individuals with DD.

Our study also aimed to investigate whether visual and phonological processing tasks could discriminate between DD and TDR group membership. The discriminant function analysis demonstrated that the digit span and the Kanji tasks were best able to discriminate between the two groups. The fact that both visual and phonological tasks were required for successful discrimination supports a position of both phonological and visual processing skills being important in the dyslexic profile. These findings represent an aspect of novelty and are better accommodated by the triangle model. Indeed, the triangle model is a domain-general model explaining reading difficulties in terms of a deficit in a tripartite of basic underlying systems (vision, phonology, and semantics). On this account, DD occurs as a consequence of damage to the phonological and visual systems, which produces difficulties in reading along with deficits in phonological or visual processing.

Taking up this point, individuals with DD performed significantly worse than TDR on the tone test in both accuracy and RTs, with large effect sizes. These findings confirmed those of previous research and indicate the presence of some low-level sensory deficit (Marshall et al., 2001; Goswami et al., 2002; Goswami, 2015). However, auditory tasks do not discriminate between the two groups when the other variables are entered into the equation. This might emphasize that, even though presenting with some low-level sensory deficit, phonological impairments explain a larger proportion of variance. Indeed, when the phonological tasks are entered into the model, they showed better discriminatory power in distinguishing between DD and TDR groups than the auditory task. Such a result stresses the importance of phonology and not merely auditory processing in adults with DD. Furthermore, it would be interesting to evaluate processing skills in the phonological and visual domains of dyslexic readers within different cultures, particularly those reading different orthographies (e.g., transparent languages such as Italian, see Provazza et al., 2019 for some considerations about dyslexia in different languages). Finally, large-scale studies should be performed to understand whether dyslexia operates as an umbrella term encompassing several different problems, such as phonological and visual processing (Giofre et al., 2019).

This study highlights some interesting future research directions. It could be argued that the visual tasks included in this study might require both visual decoding and visual perceptual processing. We are of the view that the visual deficit and visual attention deficit may be overlapping – they both recruit activations of ventral and dorsal visual pathways, but they are, at least in part, distinguishable. Visual tasks in the present study might be more related to some basic visual decoding skills rather than to visual attention span. Further research is needed to disentangle these two processes, which might reflect different underlying mechanisms. The phonological test used in this task might, in some way, reflect verbal short-term-memory capacities. In fact, it can be argued that the auditory matching tasks also involved short-term memory considering that the presentation is serial as compared to the simultaneous presentation of stimuli in visual matching tasks.

Furthermore, the VSWM tests might also be related with visual short-term memory, verbal short-term memory, and some basic visual decoding. For instance, some tasks seemed not so related to visuo-spatial attention but exhibited some relationship with memory. This could be accounted, for example, by the working memory triarchic model postulated by Baddeley and Hitch (1974), which contains both visual spatial processing and the verbal circle. This is also accommodated by other WM models such as those considering attention as a fundamental part of working memory capacity (see for example Engle, 2010 for an historical perspective). The authors recognize that it is often very hard to distinguish between attention and WM, since very simple tasks might require memory resources while at the same time complex span tasks always require higher levels of cognitive control and higher attentional resources (see Engle, 2010 on this point). Despite these limits, the evidence here raises questions about the range of possible causes of DD, including the often overlooked visual processing deficit.

This research presents with several aspects of novelty compared to previous research. If the generalized visual impairment hypothesis for DD is correct, then a number of key questions emerge including: (1) what is the critical nature of the visual impairment and (2) why are written words so vulnerable to this impairment? Answering these questions will necessitate further research using a larger sample but the present study indicates that individuals with DD are impaired on visual, phonological and visuo-spatial tasks. For this reason, we can speculate that individuals with DD may present with a deficit in the visual as well as in the phonological domain, and that their difficulties in reading may arise as the consequence of these several deficits. As expected, not all individuals with DD showed the same pattern of impairment compared to the TDR group (see Supplementary Material). These findings confirm that DD is a complex disorder characterized by deficits in different cognitive mechanisms (visual and phonological) that underpin reading. Practitioners working in this field should thus consider assessing a diverse range of abilities rather than limiting their focus to phonological skills, to fully capture the difficulties children may encounter when learning to read.

## Data Availability Statement

The datasets generated for this study are available on request to the corresponding author.

## Ethics Statement

This study was carried out in accordance with the recommendations of Liverpool John Moores University Psychology Research Ethics Committee with written informed consent from all subjects in accordance with the Declaration of Helsinki. The protocol was approved by the Liverpool John Moores University Psychology Research Ethics Committee.

## Author Contributions

Testing and data collection were performed by SP. Data analysis was performed by SP, DG, and DR. All authors contributed to the study design, interpreted results, drafted the paper, provided critical revisions, and approved the final version of the paper for submission.

### Conflict of Interest

The authors declare that the research was conducted in the absence of any commercial or financial relationships that could be construed as a potential conflict of interest.
