# Supplementary material for: Double Trouble: Visual and Phonological Impairments in English Dyslexic Readers
Source: Front Psychol. 2019 Dec 17;10:2725. doi: 10.3389/fpsyg.2019.02725 (PMC6927912; doi:10.3389/fpsyg.2019.02725)

**Supplementary material**

Graphic representation of the pattern of results shown by each participant across all tasks.

**Balloons**


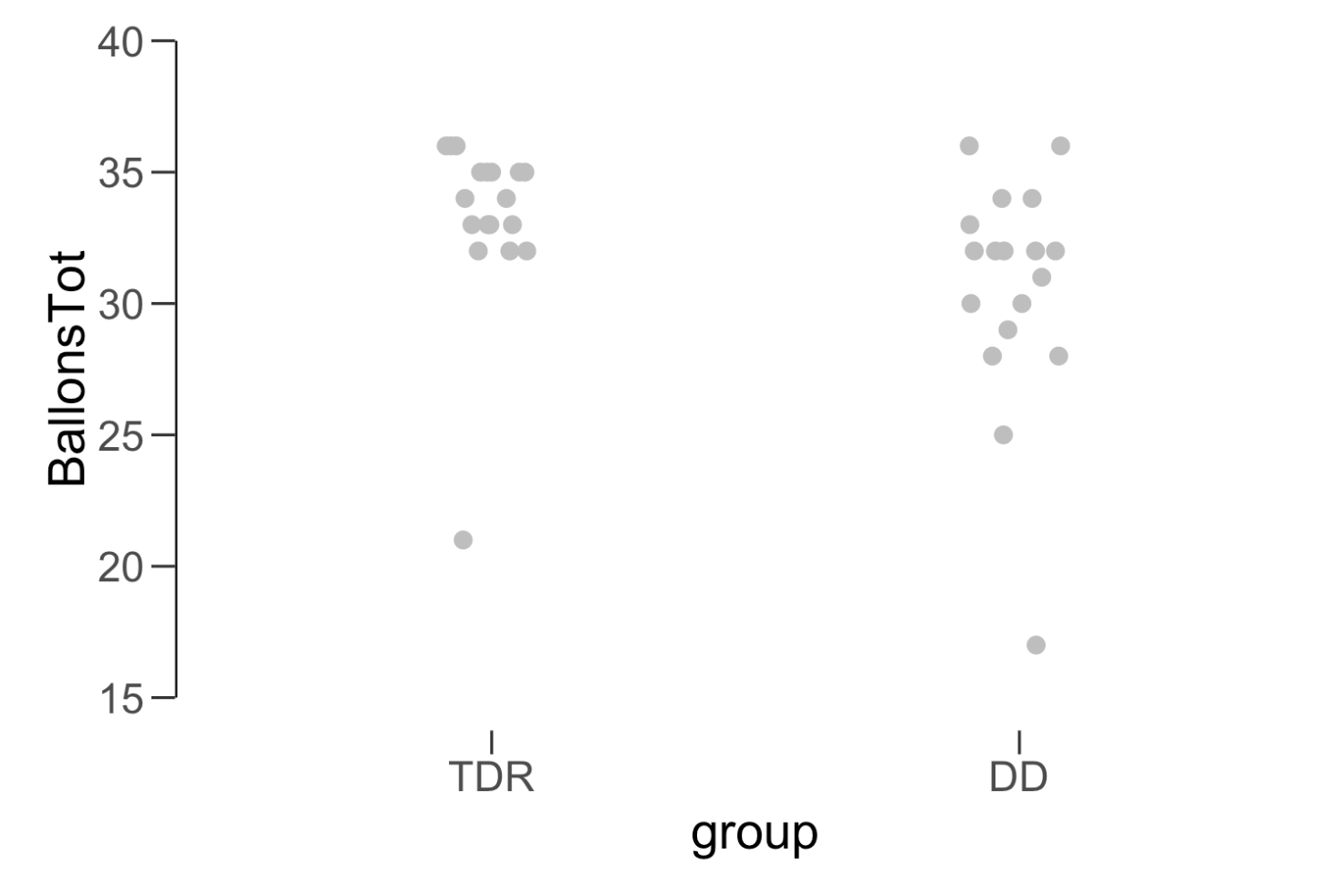


**Sequential matrices**


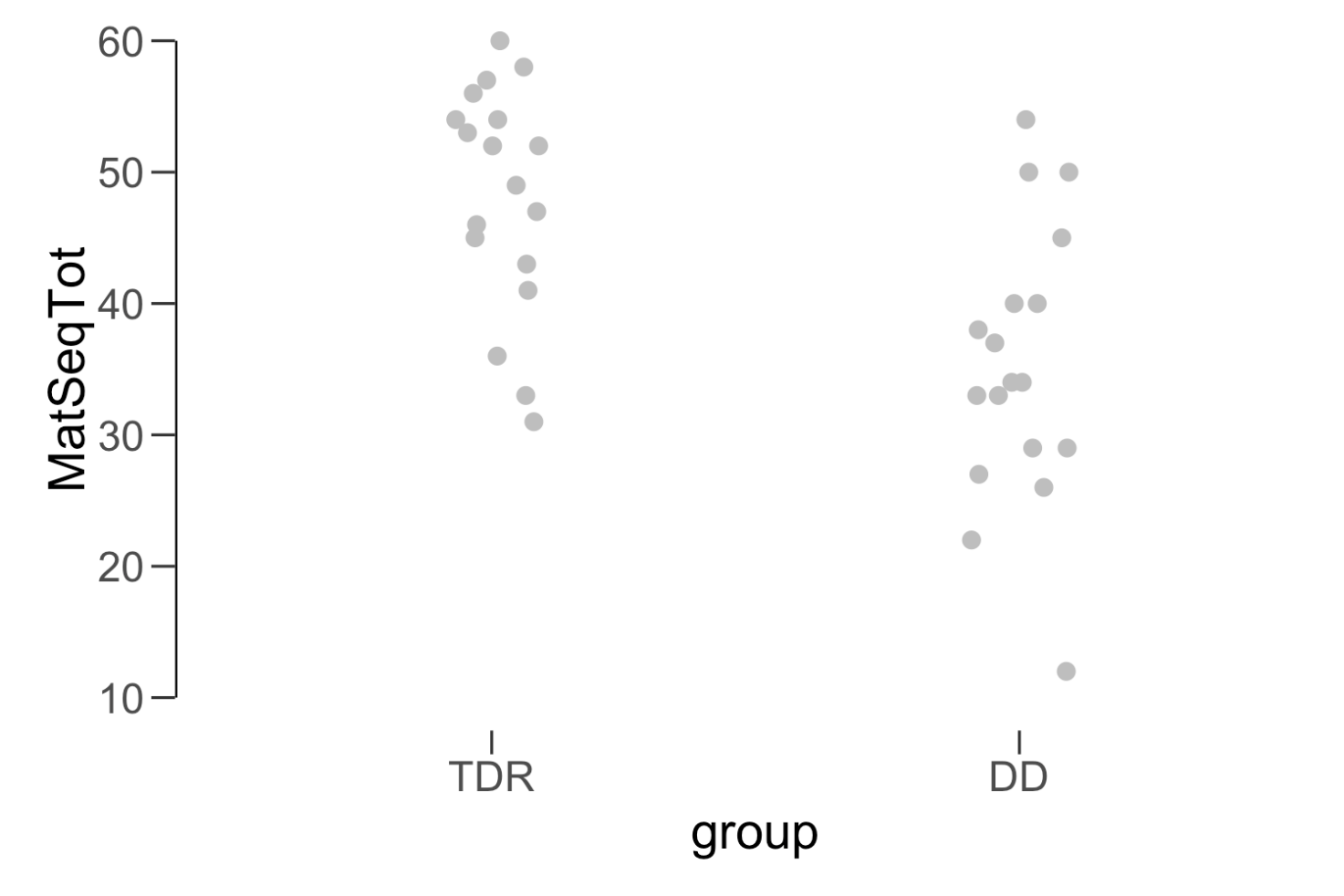


**Simultaneous matrices**


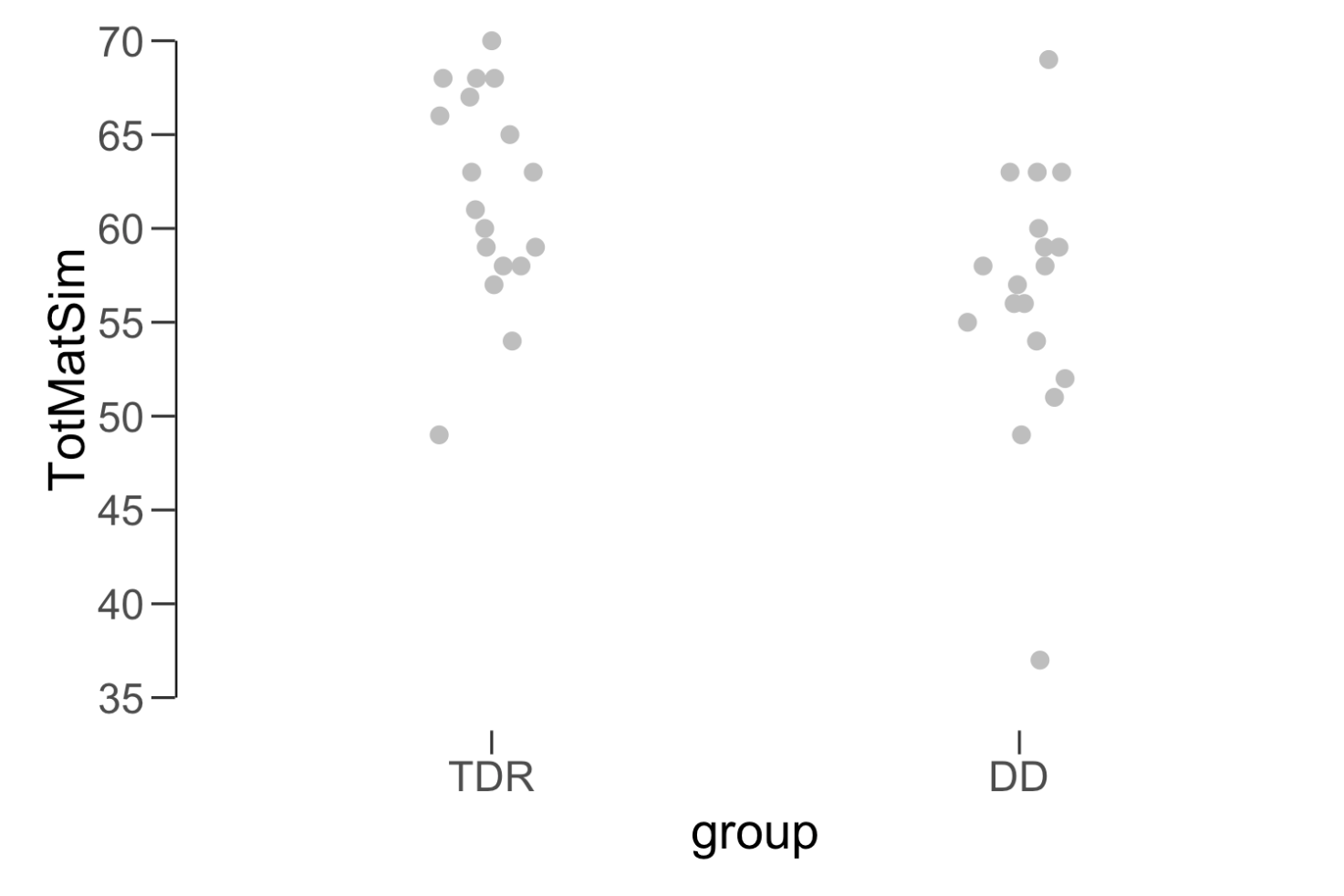


**Checkerboard reaction times**


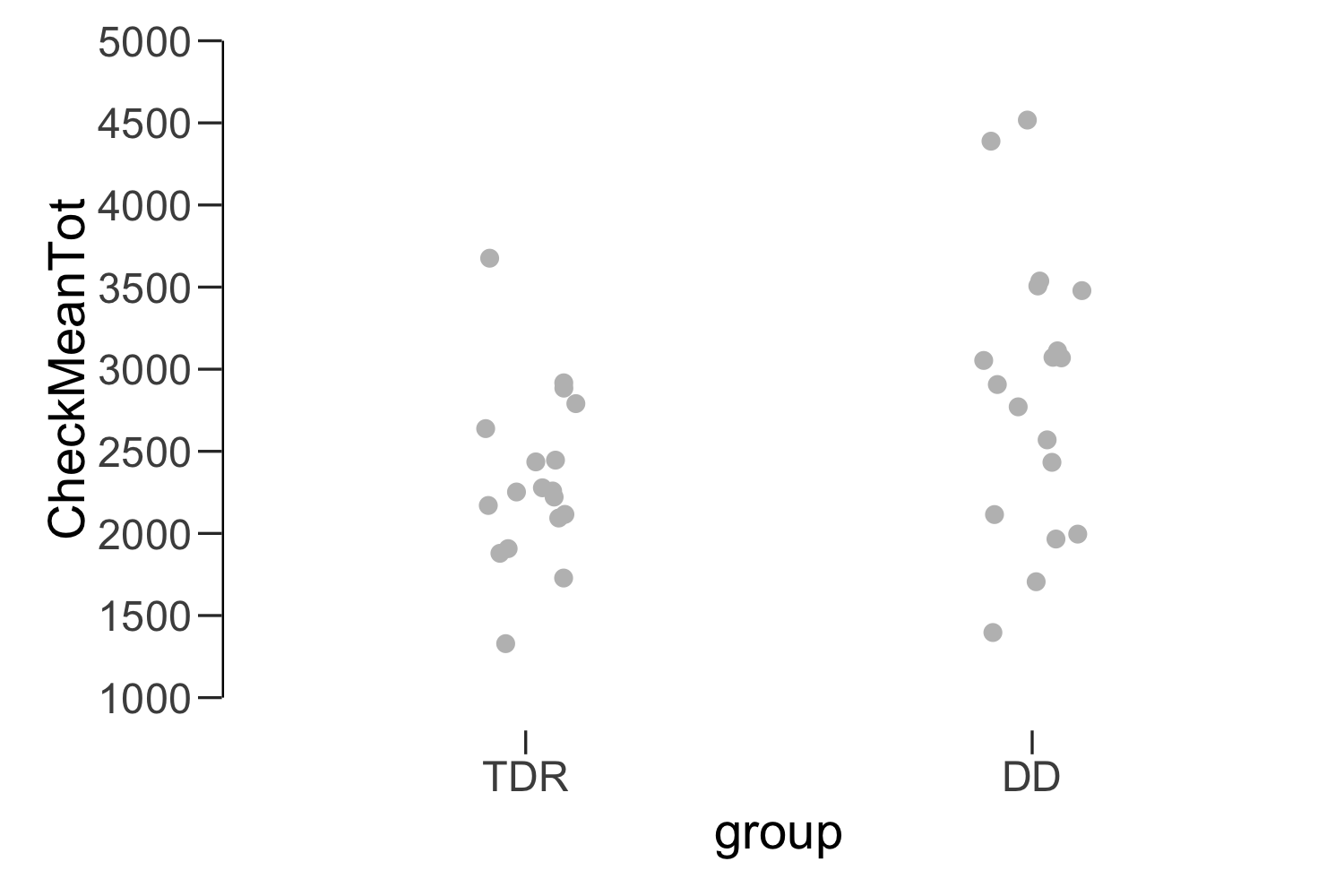


**Checkerboard accuracy**


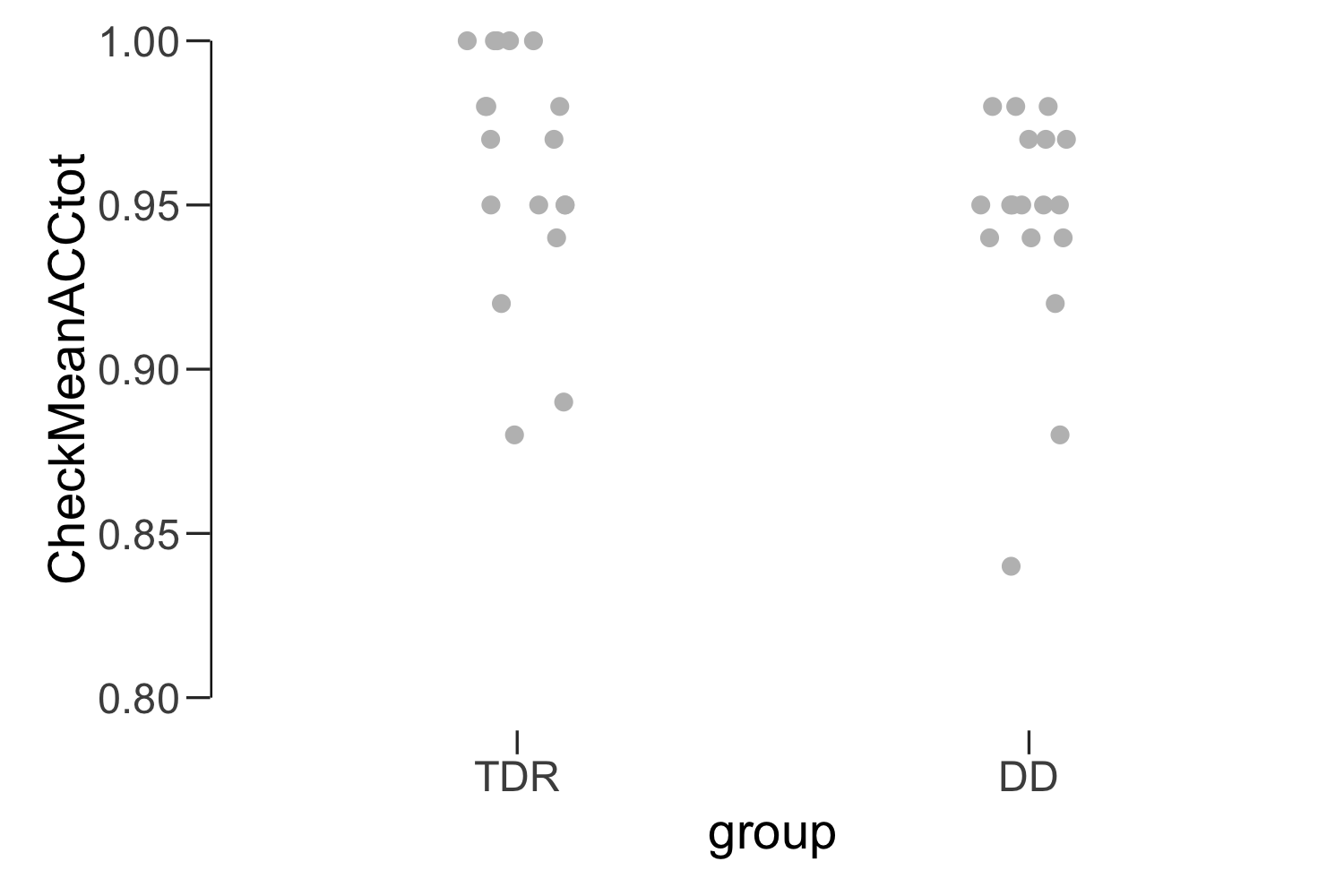


**Kanji reaction times**


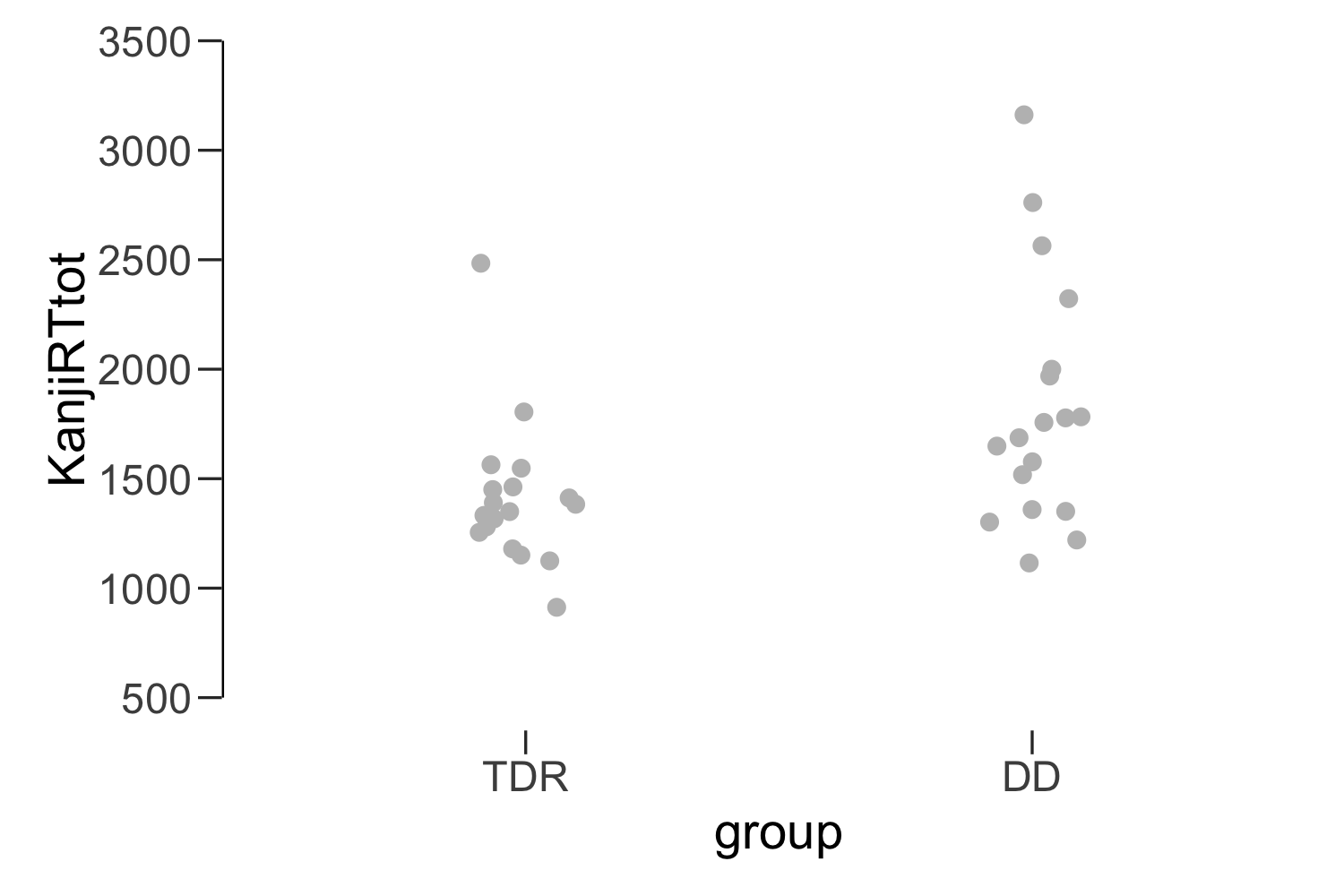


**Kanji accuracy**


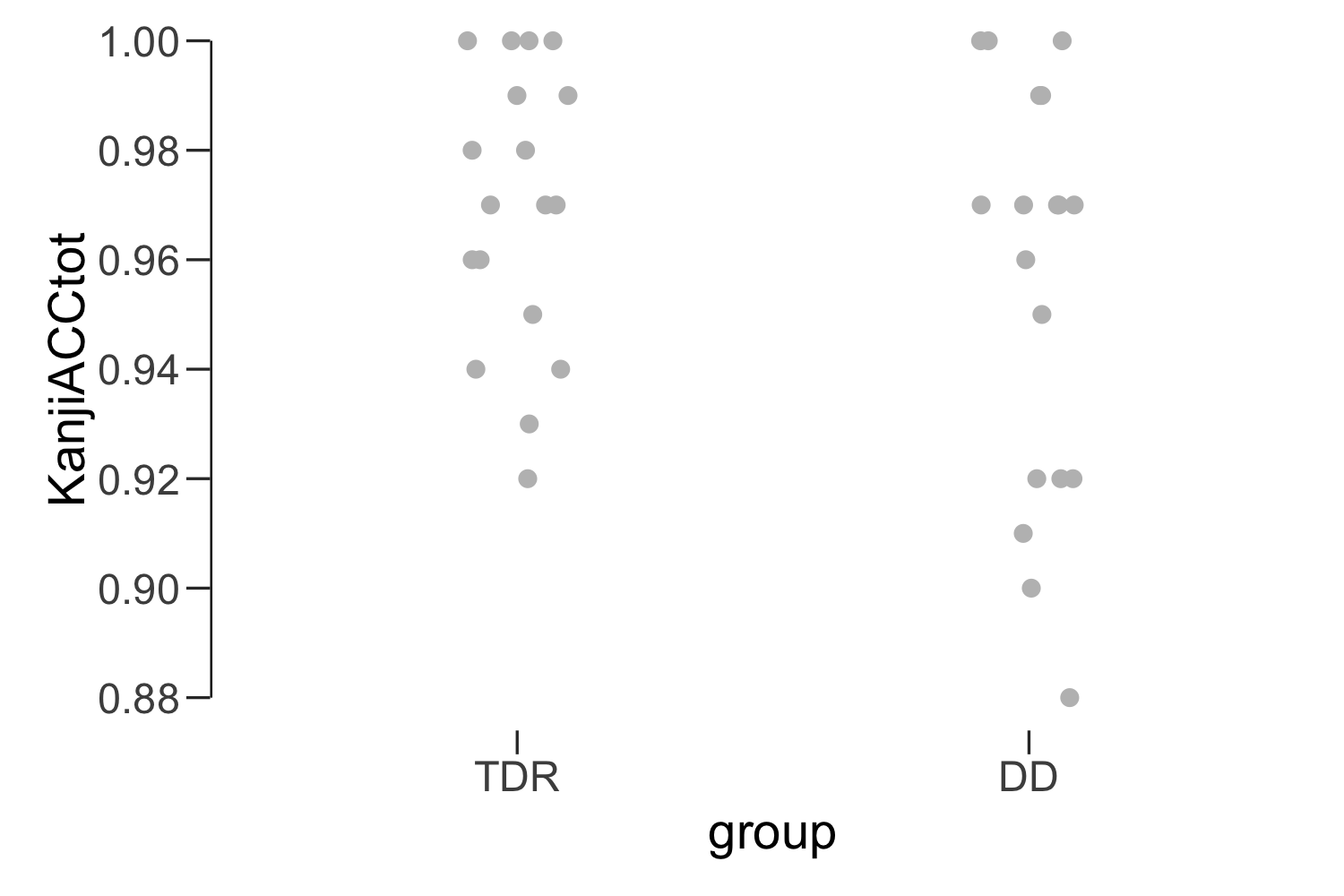


**Tone test reaction times**


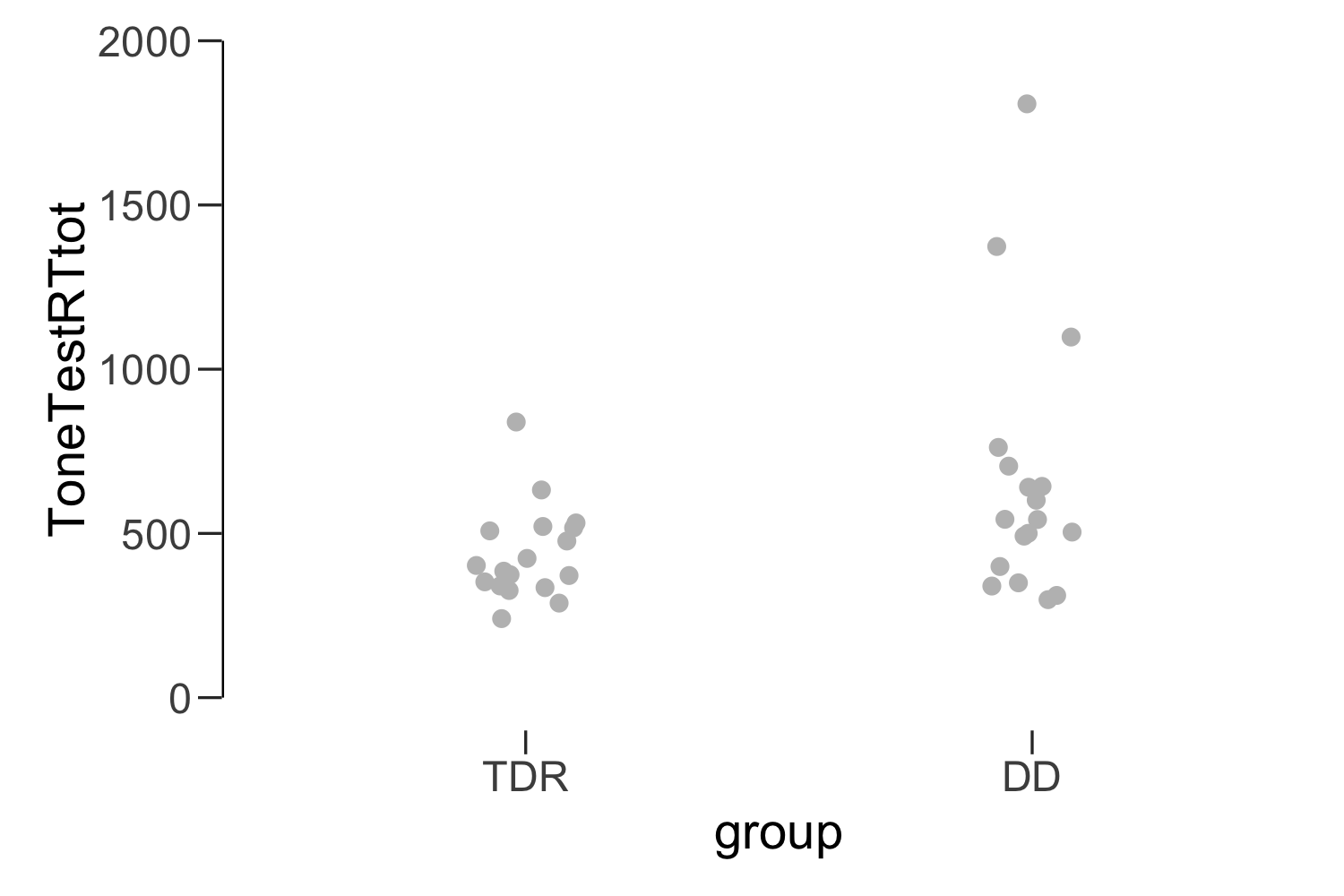


**Tone test accuracy**


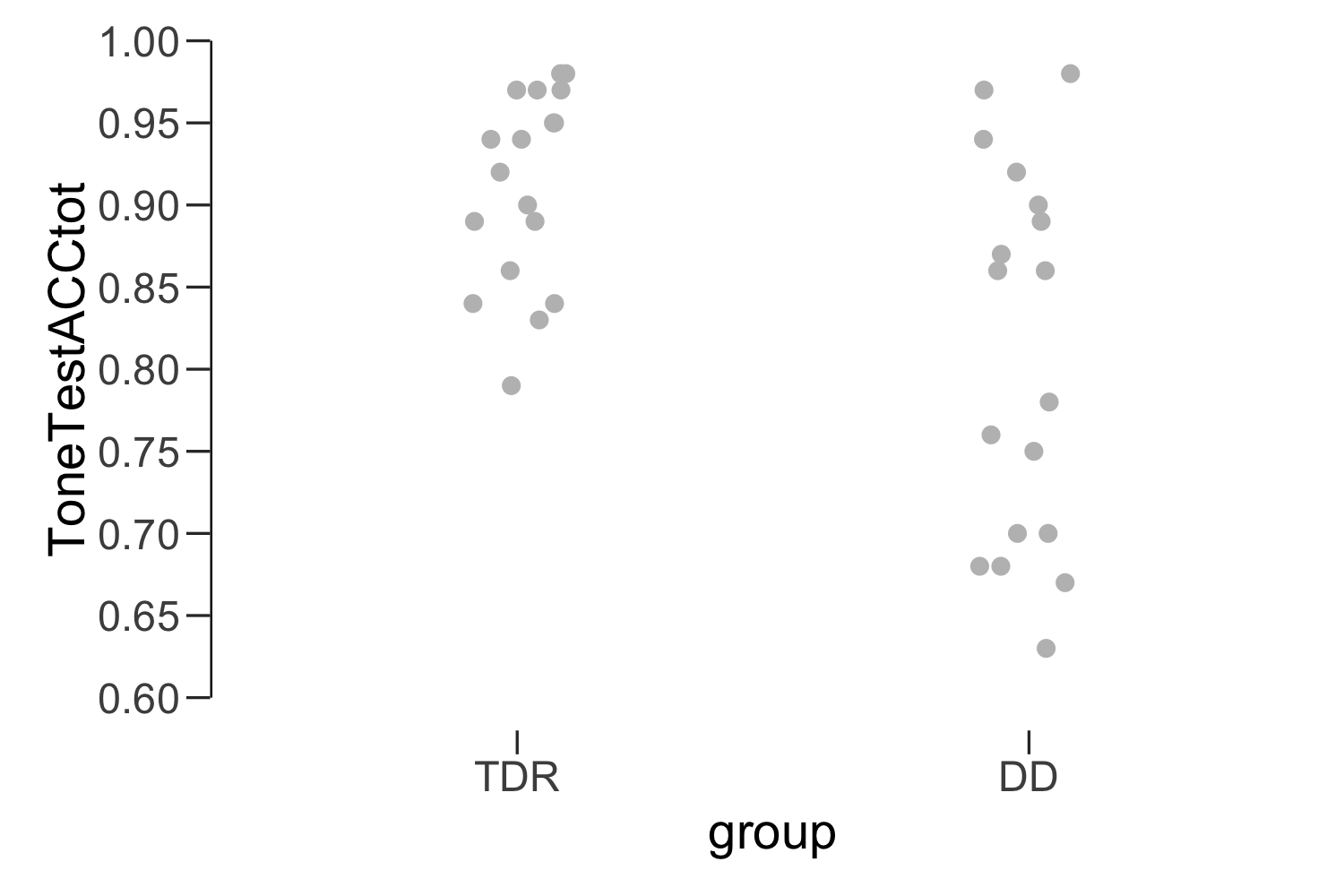


**Digit span**


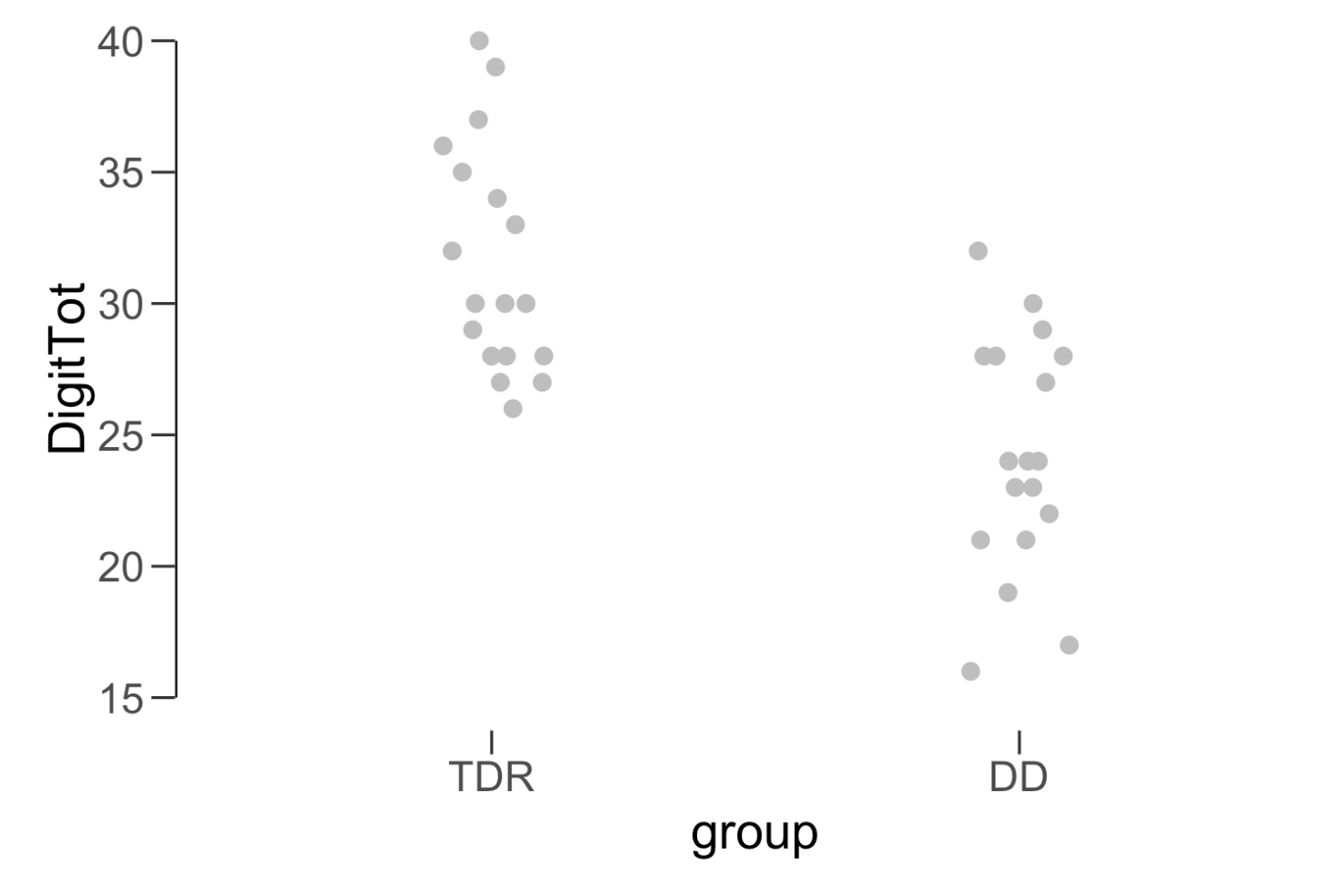

Supplement: Supplementary file 1 [file Data_Sheet_1.docx]
